# One Health Approach to Brazilian Spotted Fever: Capybaras, Horses, and Rural Areas as Predictors for Human Disease

**DOI:** 10.3390/pathogens14040305

**Published:** 2025-03-23

**Authors:** Iara Giordano Rosa-Xavier, Adriano Pinter, Rogério Giuffrida, Alexander Welker Biondo, Louise Bach Kmetiuk, Vamilton Alvares Santarém

**Affiliations:** 1 Graduate College in Animal Sciences, University of Western São Paulo (UNOESTE), Presidente Prudente 19026-310, SP, Brazil; iaragrx@yahoo.com.br (I.G.R.-X.); rgiuffrida@unoeste.br (R.G.); vamilton@unoeste.br (V.A.S.); 2 Veterinary Medicine and Zootecnic, Veterinary Medicine School, University of São Paulo, São Paulo 05508-270, SP, Brazil; adrianopinter@gmail.com; 3 Department of Veterinary Medicine, Federal University of Paraná, Curitiba 80035-050, PR, Brazil; 4 Zoonosis Surveillance Unit, City Secretary of Health, Curitiba 81265-320, PR, Brazil; louisebachk@gmail.com

**Keywords:** epidemiology, *Rickettsia*, risk factors, tick, vector-borne diseases, zoonoses

## Abstract

(1) Background: Brazilian spotted fever (BSF) is a tick-borne disease that has occurred in several Brazilian regions, caused by *Rickettsia* spp. bacteria and mainly transmitted by *Amblyomma* ticks. Despite the high BSF lethality in several Brazilian endemic areas, predictors and associated risk factors remain to be fully established. Accordingly, the retrospective study herein aimed to assess BSF cases and associated factors in an endemic area of western São Paulo state. (2) Methods: Notified cases identified by the System for Epidemiological Surveillance of São Paulo (CVE), from January 2007 to December 2021, were gathered and analyzed by Logistic Multivariate Regression (LMR) to assess potential risk factors for BSF. (3) Results: Overall, 74/1121 (6.6%; 95% CI: 5.29–8.21) individuals were considered positive for BSF. Univariate analysis showed previous contact with capybaras (OR: 1.89; 95% CI: 1.0–3.55; *p* < 0.001) and raising horses (OR = 1.4; 95% CI: 0.66–2.67; *p* = 0.45), while LMR revealed living in rural areas (OR = 2.0; 95% CI: 1.02–3.73; *p* = 0.037) as an associated risk factor for BSF. (4) Conclusions: The results herein show that the geographically studied area still shows high occurrence of BSF, mostly for individuals living or visiting areas overlapping free-ranging capybaras.

## 1. Introduction

Brazilian spotted fever (BSF) is a major tick-borne rickettsial disease in Brazil, caused by bacteria of the genus *Rickettsia* spp. and transmitted by *Amblyomma* ticks [1,2], with compulsory notification required by the Ministry of Health [3].

Human clinical features of BSF have varied from mild to severe according to rickettsial species involved and may initially include fever, headache, myalgia, and cutaneous rash [4]. Without correct clinical management, severe presentation may include neurological signs, seizures, icterus, acute renal insufficiency, hemorrhagic suffusion, and necrosis [4].

The BSF endemic areas are mainly located in southeastern Brazil, mostly in the São Paulo state, making up 44.2% of national cases. These areas have been characterized by a much higher *Amblyomma* tick burden on both capybaras (*Rickettsia rickettsii*-amplifying hosts) and in the environment, when compared to the BSF nonendemic areas [3,5]. The expansion of BSF endemic areas in São Paulo state has been associated with the increase in sugarcane cultivation areas and the increment in capybara (*Hydrochoerus hydrochaeris*) populations, a rodent that plays an important role as a *Rickettsia rickettsii* amplifier, along with maintaining *A. sculptum* [6].

In such a scenario, the Middle Paranapanema River region of São Paulo state has shown one of the highest BSF incidence (7.29/10,000 inhabitants) [7]. The expansion of agriculture, mainly sugarcane plantations, the availability of water and food sources, and the local extinction of natural capybara predators (jaguars, ocelots, caimans, and anacondas) have led to the spread of resident capybara populations in São Paulo.

Despite the importance of public health in BSF endemic areas in São Paulo state, no study, to date, has characterized the associated factors for BSF in this specific region, considering Reportable Disease Information System-SINAN, Ministry of Health. Thus, the present study aimed to investigate socio-epidemiological and environmental variables associated with suspected BSF cases from January 2007 to December 2021, which were identified, followed, confirmed, and input into the System for Epidemiological Surveillance of São Paulo (CVE).

## 2. Materials and Methods

### 2.1. Ethics Statement

The present study was approved by the Ethics Committee in Human Health of the Brazilian National Health Council (Protocol Number 62310322.8.0000.5515).

### 2.2. Study Design

This is a retrospective epidemiological study of BSF-notified cases in Middle Paranapanema River region, Brazil between January 2007 and December 2022, based on the Brazilian health epidemiological surveillance system of the Ministry of Health. This study aimed to describe the associated risk factors of BSF human cases in a BSF endemic area of Brazil.

Suspected cases were primarily confirmed by the isolation of an etiological agent (culture and/or PCR), serological testing (pared testing with 15-day interval with a 4-fold increase in seroconversion), or positive immunohistochemistry to Rickettsia sp. antigens. In addition, clinical–epidemiological criteria may be used in endemic areas, following a standard guideline of BSF diagnosis [8].

### 2.3. Study Area

**Figure 1:**
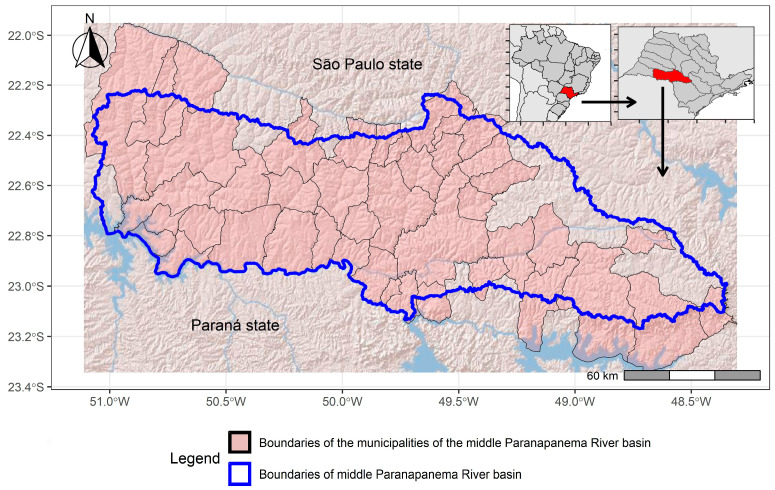
Geopolitical limits of the municipalities within the Middle Paranapanema River basin, São Paulo state, southeastern Brazil.

The present study was conducted in the Middle Paranapanema River region (Figure 1), with 25 (out of 645 statewide) municipalities distributed over 6237 km^2^, with an overall population of 465,170 inhabitants [9].

This region has been mainly covered by grassland and sugarcane cultivation, and it has been considered the main sugarcane producing region of the world [10] with a sugarcane expansion surpassing 100% (2 million hectares) over the last 15 years [11].

### 2.4. Data Source

This study was based on the information provided by the investigation [12] of BSF-notified cases in the System for Epidemiological Surveillance of São Paulo (CVE), a part of the Brazilian health epidemiological surveillance system, Ministry of Health, from Paranapanema River region, São Paulo, between January 2007 and December 2022. The information was provided by the National Disease Notification System (SINAN).

The information obtained from the database included age, ethnicity, household location, dog or cat owning, cattle raising, and horse raising. In a 14-day period before the first clinical signs, the presence of tick infestation, visits to areas with capybaras, and visits to forests were registered, in compliance with the epidemiological questionnaire used by the SINAN, Brazilian Ministry of Health. The BSF case definition adopted herein also followed the Brazilian Ministry of Health (Supplementary file S1).

The records were organized into electronic spreadsheets, with the exclusion of duplicated records.

### 2.5. Statistical Analysis

Data were categorized and submitted for the univariate analysis using the Pearson Chi-Squared Test.

A total of 1121 confirmed cases were considered for analysis due to the consistency of epidemiological information and included in the univariate analysis. Finally, due to the missing information from part of the records, only 885 individuals were subjected to logistic regression. The maximum information loss observed herein was 1.43% for the history of tick infestation.

All predictors with *p*-values below a predefined threshold (*p* < 0.20) were selected for the logistic regression model. Forward stepwise selection was adopted to determine which variables should be retained in the logistic final model. The Akaike Information Criterion (AIC) was used to evaluate improvements in the model at each step. To evaluate possible confounder variables in the final model, the predictor variables were tested for multicollinearity and exclusion of inflation factor of variance higher than 4.0 (IFV > 4.0). From the regression coefficients for each predictor variable, the odds ratio values were estimated per point and with a 95% Confidence Interval (95% CI). All analyses were performed using the broon and car packages available in the R program v.4.2.2 [12,13]. A significance level of 5% was adopted for all statistical tests [11].

Variables that presented statistical significance lower than 0.20 (*p*-value > 0.2) in the univariate analysis were subject to the multivariate analyses (logistical regression). Logistic regression was used to assess risk/protective factors for BSF. To improve the final model, the predictor variables were tested for multicollinearity and the exclusion of the inflation factor of variance higher than 4.0 (IFV> 4.0). From the regression coefficients for each predictor variable, the odds ratio values were estimated per point and with a 95% Confidence Interval (95% CI). The best-fitting model was considered the one that included significantly associated variables (*p*-value < 0.05) and minimized the Akaike Information Criterion (AIC) value.

All the statistical analyses were performed using R software v.4.2.2. A significance level of 5% was adopted for all statistical tests [14].

## 3. Results

BSF was confirmed in 74/1121 (6.6.%; 95% CI: 5.29–8.21) individuals. Most of the individuals were male (801/1121; 71.5%; 95% CI: 68.74–74.02), self-declared as white (921/1121; 82.2%; 95% CI: 79.81–84.29), and lived in urban areas (917/1121; 81.8%; 95% CI: 79.44–83.95) (Table 1), with age ranging from 1 to 85 years (median: 35) in suspected individuals, and from 3 to 75 years (median: 33) in BSF positive persons.

Univariate analysis showed that history of previous capybara contact (OR: 1.89; 95% CI: 1.0–3.55; *p* < 0.001) and raised horses (OR = 1.4; 95% CI: 0.66–2.67; *p* = 0.045) were statistically associated risk factors for BSF. The variables, (1) history of tick infestation, (2) visit to forest, and (3) living in rural area, were fitted to be included in the multivariate analyses (Table 2). One step was performed during the forward stepwise selection process, and all five variables were retained in the final logistic regression model. All five variables were retained in the final logistic regression model. The remaining variables, including age, gender, ethnicity, dog or cat owning, and living with cattle, were not considered fit (*p*-value > 0.2) to be included in the multivariate analyses.

**Figure 2:**
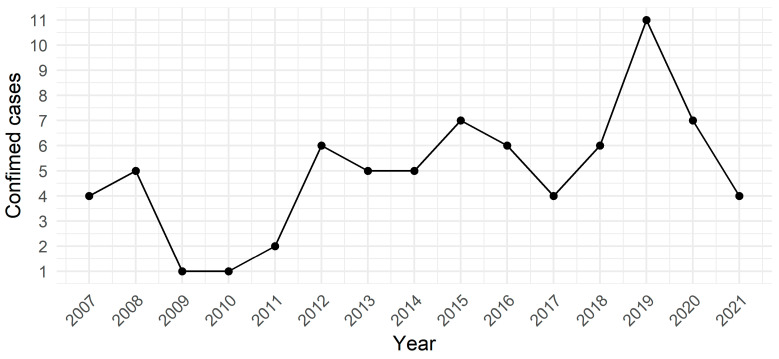
Time series of confirmed cases of Brazilian Spotted Fever in municipalities in Middle Paranapanema River basin, São Paulo state, southeastern Brazil, from 2007 to 2021.

**Figure 3:**
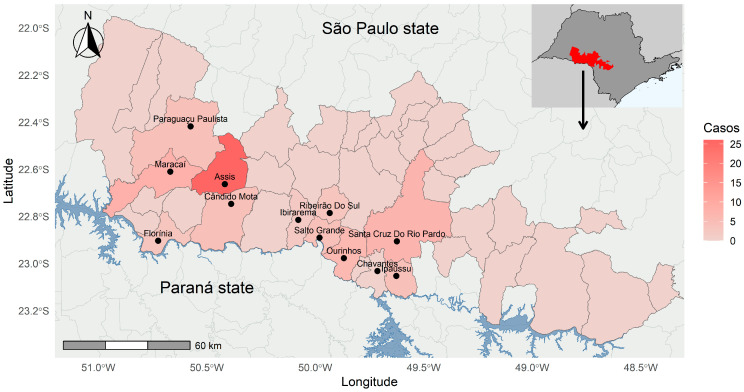
Geographic distribution of confirmed FMB cases in municipalities of the Middle Paranapanema Basin reported between 2011 and 2021 in the state of São Paulo, Brazil. The dots represent the municipal seats.

Herein, the number of confirmed cases ranged from 1 (in 2010) to 11 (2019) (Figure 2), with the highest number of cases in April (11 cases) and the lowest in July (1 case). The geographical distribution of confirmed FMB cases in the municipalities of the Middle Paranapanema Basin from 2011 to 2021 was gathered and presented (Figure 3).

## 4. Discussion

The present study evaluated the risk/protective factors of BSF, in an endemic area of Brazil, from 2007 to 2021. Overall, 74/1121 (6.6%) suspected individuals were confirmed to have BSF, reinforcing the endemicity in the Middle Paranapanema River region, as previously observed by spatial analysis (confirmed BSF cases between 2009 and 2019) that demonstrated a high incidence rate (7.29/10,000 inhabitants), particularly in the Florínea-Assis region [7]. The outcome herein was higher than 4.2% (22/525) of Pedreira county, São Paulo state [15], and lower than 12.2% (40/328) of the Paraná state coastal area, southern Brazil [16]. According to the latter authors, differences among infection rates of *Rickettsia* spp. in ticks may explain the range of positivity in endemic areas for BSF in Brazil [16].

The logistic regression of “living in rural areas” represented the unique risk factor for BSF, corroborating with the highest incidence of BSF in Brazil, which has been observed both in individuals living in rural areas who visit forest areas, rivers, and waterfalls [3], and in urban or periurban inhabitants due to the leisure or work in peri-urban areas [17]. Despite the present study not having assessed professional activity as a potential associated factor for BSF, occupational exposure and leisure activities should always be carefully considered in BSF endemic areas.

Other important variables were associated with BSF, based on the univariate analysis, including “previous visit to local frequented by capybaras”, corroborating previous findings in the Piracicaba River basin, state of São Paulo [17]. The presence of capybaras has been more frequently observed in rural areas and may be considered in the BSF transmission for the studied population. Yet, forest patches settled in rural areas have been usually associated with higher small-mammal diversity and higher tick density rates, increasing the likelihood of *Rickettsia* spp. transmission, as observed in the Brazilian Atlantic Forest biome [18].

Another variable associated with BSF herein was “raising horses”, corroborating previous findings that high *Rickettsia rickettsii* seroprevalence in horses was a risk for transmission in BSF silent areas [17]. Expectedly, capybaras and other wildlife species involved in the natural BSF cycle may impact the high equine seroprevalence and indicate a strong potential for human transmission. The results herein suggested that overlapping areas of horses and capybaras may pose a critical risk for human BSF infection.

Along with capybaras, horses have also been considered the primary hosts of all parasitic stages of *A. sculptum* in Brazil [19]. Under experimental conditions, horses have not shown competence in transmitting *Rickettsia rickettsii* to *Amblyomma sculptum* ticks and maintaining the BSF cycle in nature [20]. In addition, horses infected with *R. rickettsii* have shown detectable IgG antibodies with high titers and long-lasting persistence, with no clinical signs [20]. Adults and nymphs of *A. sculptum* have also been reported in cattle and several other domestic and wildlife species. Despite overlapping areas with free-range capybaras, particularly in pastures nearby water bodies, cattle may not be able to sustain *A. sculptum* populations as primary hosts [21].

Although “previous tick infestation” was not associated with BSF, such result should be analyzed with caution due to 13% (145/1121) of missing information with regard to this variable. The loss of information and quality of investigation in database, or even the notice of ticks by patients, were previously indicated to justify the difficulty in analyzing BSF’s association with tick infestation [5]. Thus, the permanent monitoring of human parasitism by ticks may provide a better understanding of tick and tick-borne disease eco-epidemiology and the early identification of potential cases of tick-borne diseases, particularly in endemic regions of spotted fever [22]. As Brazil has a wide diversity of ticks associated with human infestation and BSF cases, tick distribution may indicate endemic areas of disease. In the southern region, for instance, cases of human parasitism were more predominantly caused by *A. sculptum* in the Paraná state [23], while *A. parkeri* was more dominant in the neighboring Rio Grande do Sul state [22]. Other less frequent tick species, compared to the *A. oblongoguttatum*, have also been considered a potential factor of SFG in the Amazon Savannah of the Rondônia state, northwestern Brazil [24].

Despite BSF cases being associated with age [25] and gender [5], no statistical significance was found herein for age, gender, and ethnicity. As previously stated, the low number of positive cases may have not been enough to statistically detect differences in exposure and predictive factors for BSF [16].

The Middle Paranapanema River is a borderline between Western São Paulo state and Northern Paraná state, southern Brazil, presenting most death cases, while mild BSF cases and hospitalizations were located at the Paraná state, based on a previous epidemiological study from January 2006 to December 2017 [26]. Therefore, Paraná state’s influence on BSF occurrence in the Middle Paranapanema River should be further investigated, particularly due to capybara cross-state migrations and human leisure and work activities.

Herein, the highest number of BSF occurrences was presented in April 2019, with 11 reported cases. This result contrasted with previous reports that showed BSF cases occurring mainly during the winter and spring months, between June and October in Brazil [27]. This period was recognized for the predominant presence of nymphs, which have shown higher aggressiveness toward humans, smaller size than adult ticks (which may avoid awareness), and wider spread over the infested skin area [28]. Thus, the authors speculate that 2019 was an atypical year, with an earlier nymph season peak (or earlier higher BSF infecting transmission), leading to the highest case load about 2–3 months prior to BSF pattern.

Although current guidelines such as STROBE [29] may have improved the reporting of the present epidemiological study, this statement has been focused on cohort, case–control, and cross-sectional studies, which were not used herein. The Brazilian National Digital Health Strategy is a current effort for e-Health establishment in the Brazilian Unified Health System (UHS). This initiative comprehended an eight-year (2020–2028) set of actions to improve the UHS database nationwide. The expected benefits included service improvement for access to healthcare information, reliability and security improvement in patient information, more accuracy of diagnosis and rapid access to patient information nationwide, improvement in transparency, and citizen empowerment. Such improvement in the nationwide system may positively impact the notification, report, diagnosis, control, and prevention of Brazilian spotted fever (BSF) and other important zoonotic diseases. Although still theorical, a BSF notification system capable of gathering human, animal, and environmental (tick) information and serological/molecular testing would provide a true One Health approach to disease.

The limitations in the present study included the under-reporting of cases in the epidemiological surveillance systems and the low number of completed questionnaires in database records. Such limitations have been previously indicated in an eco-epidemiological study of BSF in Brazil [3], indicating that the national notification system should make an effort for better BSF case reporting. In addition, information given by patients, including visits to forest, contact with capybaras, horses, and tick infestation may have been biased due to answer subjectivity. Another limitation was the scarce number of cases over the investigated timeline, which did not provide sufficient information for modeling a temporal series of confirmed BSF cases. Also, contact or interaction with horses and capybaras was not included in the national surveillance database and consequently not tested herein, limiting the analysis and results. The notification system should be updated and include such questions, which would provide important information for BSF epidemiology, control, and prevention

## 5. Conclusions

In conclusion, the study herein has shown that the geographically studied area still demonstrates high BSF occurrence, mostly for individuals living or visiting areas overlapping free-ranging capybaras, reinforcing the importance of avoiding tick exposure and capybara proximity for BSF prevention in such endemic rural areas. As horses, capybaras, and ticks have shown important roles in human BSF epidemiology, a notification system capable of gathering human, animal, and environmental (tick) information, with human–animal–tick serological and molecular testing, would provide a true One Health approach to disease.

## Figures and Tables

**Table 1:** Associated factors for Brazilian Spotted Fever (BSF) cases in 1121 suspected cases in the Middle Paranapanema River basin, São Paulo state, southeastern Brazil, from 2007 to 2021. The case confirmation followed the epidemiological surveillance adopted by the Health Ministry of Brazil (Supplementary file S1).

	Suspected Cases		Univariate Analysis	
	Confirmed (%)	Not Confirmed (%)	Odds Ratio (95% CI)	*p*-Value
Variables	74 (6.6)	1047 (93.4)		
Age	*n* = 1121			0.563
1 to 18	17 (23.0)	256 (24.5)	1	
19 to 35	24 (32.4)	291 (27.8)	1.24 (0.65–2.40)	
36 to 51	14 (18.9)	265 (25.3)	0.80 (0.38–1.66)	
52 to 85	19 (25.7)	235 (22.4)	1.22 (0.61–2.43)	
Gender	*n* = 1121			1.0
Female	21 (28.4)	299 (28.6)	1	
Male	53 (71.6)	748 (71.4)	1.00 (0.60–1.73)	
Ethnicity	*n* = 1102			0.824
White	59 (81.9)	862 (83.7)	1	
Non-white	13 (18.1)	168 (16.3)	1.14 (0.59–2.07)	
Household location *	*n* = 1108			0.033
Urban	55 (74.3)	882 (84.5)	1	
Rural	19 (25.7)	162 (15.5)	1.89 (1.06–3.22)	
Previous tick infestation *	*n* = 976			0.073
No	21 (33.3)	418 (45.8)	1	
Yes	42 (66.7)	495 (54.2)	1.68 (0.99–2.94)	
Previous capybara contact *	*n* = 984			0.001
No	35 (59.3)	731 (79.0)	1	
Yes	24 (40.7)	194 (21.0)	2.59 (1.48–4.44)	
Dog or cat owner	*n* = 996			0.731
No	30 (48.4)	481 (51.5)	1	
Yes	32 (51.6)	453 (48.5)	1.13 (0.67–1.90)	
Cattle raising				0.671
No	48 (81.4)	781 (84.3)	1	
Yes	11 (18.6)	145 (15.7)	1.25 (0.60–2.38)	
Previous visit to forest *	*n* = 985			0.064
No	10 (15.4)	244 (26.6)	1	
Yes	55 (84.6)	673 (73.4)	1.97 (1.03–4.18)	
Horse raising *	*n* = 986			0.045
No	44 (73.3)	779 (84.1)	1	
Yes	16 (26.7)	147 (15.9)	1.94 (1.03–3.47)	

* Variables fitted to the multivariate analyses. Age intervals were categorized by the 25% percentile.

**Table 2:** Associated factors for Brazilian Spotted Fever (BSF) subjected to the multivariate analyses (logistical regression), considering 1121 suspected cases in the Middle Paranapanema River basin, São Paulo state, southeastern Brazil, from 2007 to 2021.

Coefficients	β Estimate	*p*-Value	Odds Ratio (95% CI)
Intercept	−3.406	0.361	NA
Rural household location	0.687	0.037	2.0 (1.02–3.72)
Previous tick infestation	0.637	0.665	1.15 (0.62–2.16)
Previous capybara contact	0.637	0.051	1.9 (1.0–3.57)
Previous visit to forest	0.168	0.683	1.18 (0.55–2.78)
Horse raising	0.313	0.375	1.37 (0.66–2.67)

## Data Availability

The raw data supporting the conclusions of this article will be made available by the authors upon request.

## Supplementary Materials

The following supporting information can be downloaded at: https://www.mdpi.com/article/10.3390/pathogens14040305/s1. Supplementary file S1—Operational case definition adopted by the Ministry of Health for epidemiological surveillance of BSF in Brazil [8].
