# Supplementary material for: One Health Approach to Brazilian Spotted Fever: Capybaras, Horses, and Rural Areas as Predictors for Human Disease
Source: Pathogens. 2025 Mar 23;14(4):305. doi: 10.3390/pathogens14040305 (PMC12030754; doi:10.3390/pathogens14040305)
Supplement: Supplementary file 1 [file pathogens-14-00305-s001.zip › pathogens-3461492-supplementary.pdf]

Supplementary file S1. Operational case definition adopted by the Ministry of Health for epidemiological surveillance of BSF in Brazil (26).

1. Suspected Case

A person with sudden-onset fever, headache, and myalgia, associated with at least one of the following conditions: history of tick bite/tick removal OR contact with dogs and cats that have access to forested areas OR residence in/recent visit (within the last 14 days) to an area of transmission or risk for spotted fever.

OR

A person with sudden-onset fever, headache, and myalgia associated with at least one of the following conditions: appearance of a maculopapular rash between the second and fifth days of illness OR hemorrhagic manifestations, provided other causes have been excluded.

Note: If there is a significant increase in the number of cases in a specific region, the definition of a suspected case may be expanded to increase sensitivity and case detection.

2 Confirmed Case

a) Laboratory Criteria

A case with a clinical presentation compatible with spotted fever and epidemiological background, with any of the following laboratory results:

- Isolation of *Rickettsia* in culture;

- Detectable polymerase chain reaction (PCR) performed at the Adolfo Lutz Institute;

- Positive immunohistochemistry (IHC) for *Rickettsia*;

- Indirect immunofluorescence assay (IFA) showing at least a fourfold increase in titer between two samples collected 14 to 21 days apart conducted at the Adolfo Lutz Institute, the reference laboratory in the state.

b) Clinical–Epidemiological Criteria

The criteria only apply to cases with a clinical presentation compatible with spotted fever and an epidemiological background where no sample was collected for testing at an appropriate time (does not apply to cases where testing was performed and yielded negative/non-reactive results), provided there is an epidemiological link to a laboratory-confirmed case. An epidemiological link is defined as having been in a location where at least one laboratory-confirmed case of spotted fever occurred within a 15-day period.

3 Discarded Case

A case with a confirmed diagnosis of another disease or where the information obtained during the investigation (including laboratory test results) is insufficient to confirm the diagnosis of spotted fever.
